# Clinical Trials for Treatment and Prevention of HIV-Associated Malignancies in Sub-Saharan Africa: Building Capacity and Overcoming Barriers

**DOI:** 10.1200/GO.20.00153

**Published:** 2020-07-22

**Authors:** Lilie L. Lin, David S. Lakomy, Elizabeth Y. Chiao, Robert M. Strother, Meg Wirth, Ethel Cesarman, Margaret Borok, Naftali Busakhala, Carla J. Chibwesha, Lameck Chinula, Ntokozo Ndlovu, Jackson Orem, Warren Phipps, Vikash Sewram, Samantha L. Vogt, Joseph A. Sparano, Ronald T. Mitsuyasu, Susan E. Krown, Satish Gopal

**Affiliations:** ^1^ Department of Radiation Oncology, The University of Texas MD Anderson Cancer Center, Houston, TX; ^2^ Dartmouth Geisel School of Medicine, Hanover, NH; ^3^ Department of General Oncology, The University of Texas MD Anderson Cancer Center, Houston, TX; ^4^ Department of Epidemiology, The University of Texas MD Anderson Cancer Center, Houston, TX; ^5^ Department of Medicine, University of Otago, Christchurch, New Zealand; ^6^ The Emmes Corporation, Callaway, MD; ^7^ Department of Pathology and Laboratory Medicine, Weill Cornell Medical College, New York, NY; ^8^ Department of Medicine, University of Zimbabwe College of Health Sciences, Harare, Zimbabwe; ^9^ Department of Pharmacology and Toxicology, Moi University School of Medicine, Eldoret, Kenya; ^10^ Institute for Global Health and Infectious Diseases, University of North Carolina, Chapel Hill, NC; ^11^ Clinical HIV Research Unit, Department of Medicine, University of the Witwatersrand Faculty of Health Sciences, Johannesburg, South Africa; ^12^ Division of Global Women’s Health, Department of Obstetrics and Gynecology, University of North Carolina School of Medicine, Chapel Hill, NC; ^13^ UNC Project-Malawi, Lilongwe, Malawi; ^14^ Department of Radiology, University of Zimbabwe College of Health Sciences, Harare, Zimbabwe; ^15^ Uganda Cancer Institute, Kampala, Uganda; ^16^ Department of Medicine, University of Washington, Seattle, WA; ^17^ Fred Hutchinson Cancer Research Center, Seattle, WA; ^18^ African Cancer Institute, Faculty of Medicine and Health Sciences, Stellenbosch University, Stellenbosch, South Africa; ^19^ Department of Medical Oncology, Johns Hopkins Sidney Kimmel Comprehensive Cancer Center, Baltimore, MD; ^20^ Montefiore-Einstein Cancer Center, Montefiore Medical Center, Bronx, NY; ^21^ Center for Clinical AIDS Research and Education, University of California, Los Angeles, Los Angeles, CA; ^22^ AIDS Malignancy Consortium, New York, NY; ^23^ Center for Global Health, National Cancer Institute, Rockville, MD

## Abstract

**PURPOSE:**

The aim of this study was to review the current status of clinical trials for HIV-associated malignancies in people living with HIV in sub-Saharan Africa (SSA) and efforts made by the AIDS Malignancy Consortium (AMC) to build capacity in SSA for HIV malignancy research.

**METHODS:**

All malignancy-related clinical trials in 49 SSA countries on ClinicalTrials.gov were reviewed and evaluated for inclusion and exclusion criteria pertaining to HIV status. Additional studies by AMC in SSA were compiled from Web-based resources, and narrative summaries were prepared to highlight AMC capacity building and training initiatives.

**RESULTS:**

Of 96 cancer trials identified in SSA, only 11 focused specifically on people living with HIV, including studies in Kaposi sarcoma, cervical dysplasia and cancer, non-Hodgkin lymphoma, and ocular surface squamous neoplasia. Recognizing the increasing cancer burden in the region, AMC expanded its clinical trial activities to SSA in 2010, with 4 trials completed to date and 6 others in progress or development, and has made ongoing investments in developing research infrastructure in the region.

**CONCLUSION:**

As the HIV-associated malignancy burden in SSA evolves, research into this domain has been limited. AMC, the only global HIV malignancy-focused research consortium, not only conducts vital HIV-associated malignancies research in SSA, but also develops pathology, personnel, and community-based infrastructure to meet these challenges in SSA. Nonetheless, there is an ongoing need to build on these efforts to improve HIV-associated malignancies outcomes in SSA.

## INTRODUCTION

In the past several decades, major progress has been achieved for HIV prevention, treatment, and care worldwide. Today, people living with HIV (PLWH) who receive antiretroviral therapy (ART) can achieve life expectancies similar to those of HIV-negative individuals, even in low- and middle-income countries (LMICs).^1,2^ In the past decade, global ART scale-up efforts have slowed new HIV infections and halved the mortality rate among PLWH.^3^ Consequently, the global population of PLWH has never been larger.^3^ These achievements have resulted in a new global challenge: increased comorbid noncommunicable diseases among PLWH, including HIV-associated malignancies (HIVAM).^4^

**CONTEXT**

**Key Objective**
To review the current context of HIV-associated malignancies in sub-Sahara-Africa and the role of the AIDS Malignancy Consortium in advancing HIV malignancy research in this region.
**Knowledge Generated**
Only a paucity of cancer trials in sub-Saharan Africa allow for, or specifically target, those with HIV. The AIDS Malignancy Consortium has worked to establish both research infrastructure and community-based connections to better facilitate HIV-associated malignancy research in this region of the world, with particular emphasis on Kaposi sarcoma, cervical cancer, and lymphoma.
**Relevance**
As sub-Saharan Africa constitutes the highest burden of HIV-associated malignancies, greater efforts to facilitate research are needed. Whereas challenges, including inadequate diagnostic and treatment infrastructure as well as limited personnel, remain, the AIDS Malignancy Consortium has shown that HIV-associated malignancy clinical trials can be performed successfully among this population.

Sub-Saharan Africa (SSA) is disproportionally affected by HIV, accounting for approximately 70% of the global HIV burden.^3,5^ In resource-rich nations, malignancy is becoming a leading cause of mortality for PLWH,^6-8^ and there is increasing evidence that similar trends are occurring in SSA and other LMICs.^9^ HIVAM have traditionally been divided into 2 categories: AIDS-defining cancers (ADC), which include Kaposi sarcoma (KS), non-Hodgkin lymphoma (NHL), and invasive cervical cancer^10^; and non-ADCs, which include Hodgkin lymphoma and cancers of the anus, liver, lung, and head and neck, among others.^11,12^ Regardless of cancer type, PLWH experience higher overall and cancer-specific mortality than HIV-negative patients.^13,14^

Given the lack of high-quality data on HIVAM and the routine exclusion of PLWH from many cancer clinical trials, the National Cancer Institute established the AIDS Malignancy Consortium (AMC), a multicenter clinical trials group charged with investigating the optimal treatment and prevention of cancers in PLWH.^15^ Originally constituted as a United States–based effort in 1994, the mission of AMC is to study the pathobiology of malignant and premalignant disease in PLWH and to evaluate new treatment and prevention approaches with the ultimate goal of establishing better standards of care for both premalignant and malignant diseases that affect PLWH both domestically and internationally. It is with that goal in mind that AMC began to incorporate clinical trials sites in SSA in 2010 and has since conducted several clinical trials in SSA, with additional trials currently enrolling or opening soon. Here, we describe both AMC- and non–AMC-sponsored HIVAM trials in SSA, highlight AMC efforts to build capacity for cancer clinical trials, and outline future opportunities for research, clinical care, and capacity building to address HIVAM in SSA.

## SEARCH METHODS

**FIG 1:**
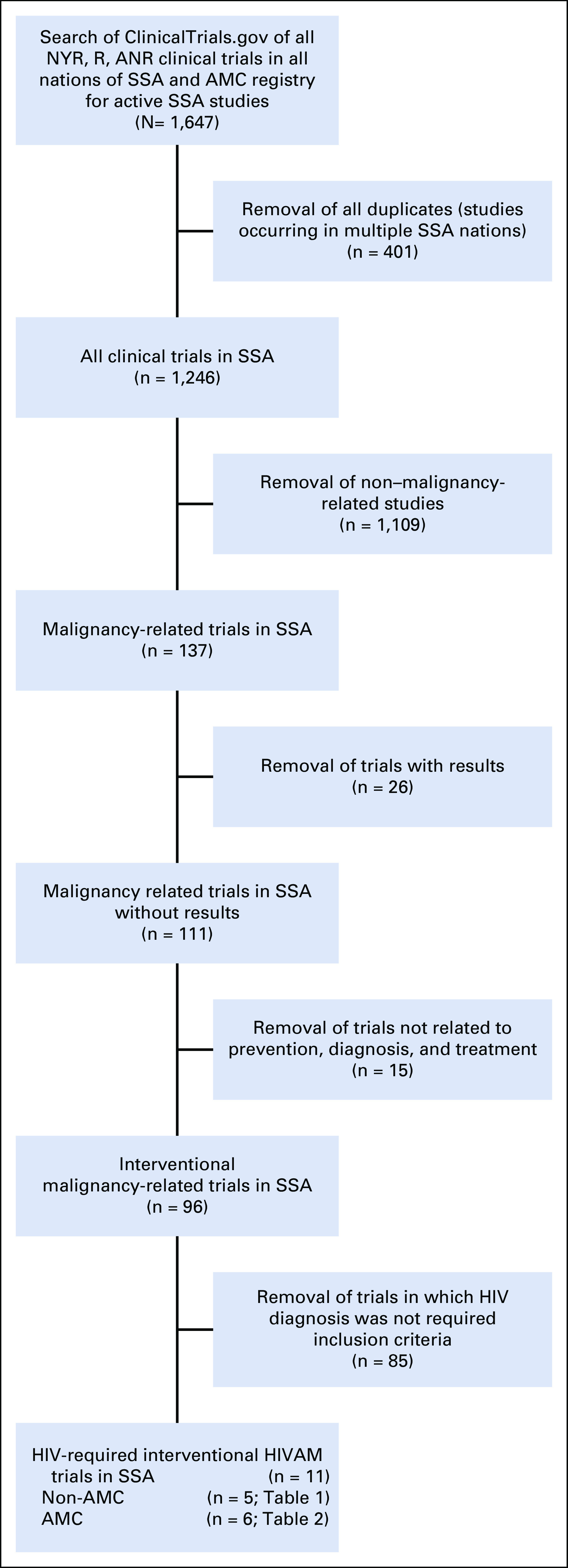
Search methodology for active cancer trials in sub-Saharan Africa (SSA). AMC, AIDS Malignancy Consortium; ANR, active, not recruiting; NYR, not yet recruiting; R, recruiting.

To assess the scope of active (ie, planned and ongoing) HIVAM-specific trials being conducted in SSA, we used the US National Library of Medicine Clinical Trials registry as well as the AMC Web site^15^ (Fig 1). To acquire a list of all active clinical trials in SSA, we searched ClinicalTrials.gov for all studies in each country in SSA as of February 19, 2020. The search was limited to studies whose recruitment status was listed as not yet recruiting, recruiting, or active not recruiting (active not recruiting studies with posted results were excluded). To focus on malignancy-specific trials, each individual study was preliminarily assessed for relevance to cancer. To gather interventional trials from this compiled list, studies were limited to prospective trials that were designed to evaluate biomedical interventions for HIVAM prevention, treatment, or supportive care. Studies that focused on behavioral interventions, epidemiologic reports, or prognostic factors were excluded.

In addition to the active trial search conducted above, completed AMC trials were identified from the AMC Web site and their corresponding publications.

## HIVAM TRIALS IN SSA

**FIG 2:**
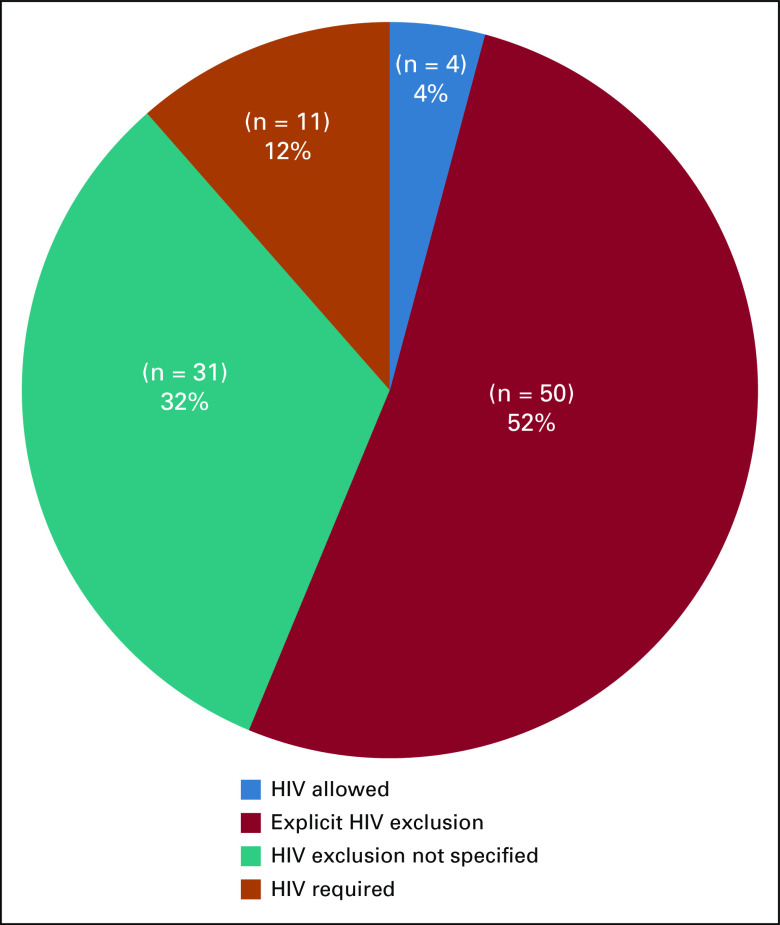
HIV eligibility of active interventional cancer clinical trials in sub-Saharan Africa.

We identified a total of 96 active SSA cancer trials through our search methods. Of these, 4 were for KS, 8 for NHL, 17 for cervical dysplasia or carcinoma, and 67 for other malignancies. Half of the trials explicitly exclude PLWH and another one third did not list HIV-specific criteria (Fig 2). Few trials allowed PLWH to enroll (n = 4; 4%), whereas 11 trials (12%) were intended specifically for PLWH. Of these 11 trials, 5 were non-AMC trials (Table 1) and 6 were AMC sponsored (Table 2).

**TABLE 1:**
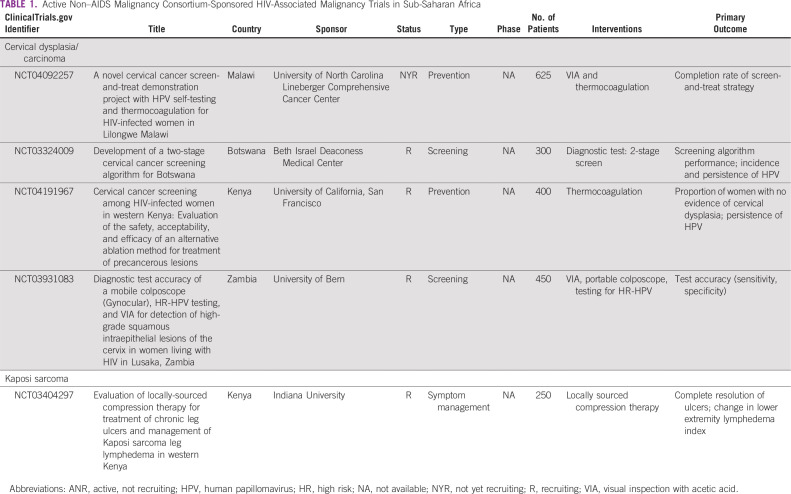
Active Non–AIDS Malignancy Consortium-Sponsored HIV-Associated Malignancy Trials in Sub-Saharan Africa

**TABLE 2:**
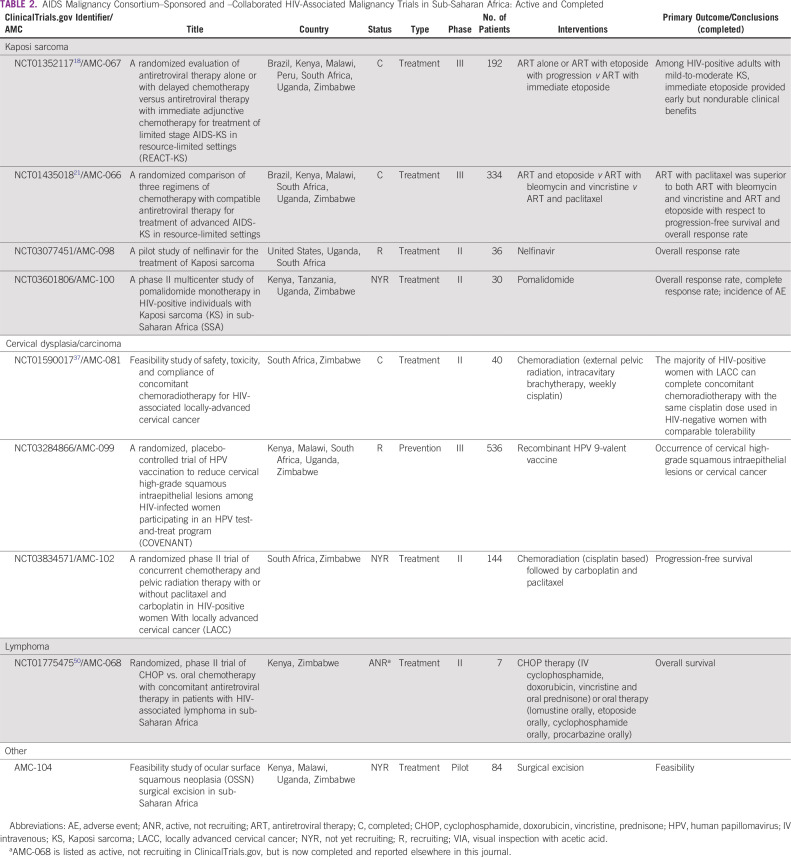
AIDS Malignancy Consortium–Sponsored and –Collaborated HIV-Associated Malignancy Trials in Sub-Saharan Africa: Active and Completed

The following sections provide a brief overview of HIVAM in SSA and describe relevant clinical trials.

### KS

Whereas KS incidence has significantly decreased with the advent of effective ART, it remains the most prevalent cancer in many SSA countries.^9^ Similar to other ADCs, KS is caused by a virus, known as KS-associated herpesvirus (KSHV) or human herpesvirus-8.^16^ Treatment of HIV-associated KS requires HIV control with ART; whereas limited-stage KS may regress with ART alone, advanced-stage KS generally also requires chemotherapy. Although KS often regresses with treatment, responses are rarely complete and treatment is seldom curative.^17^

The only non–AMC-sponsored KS study we identified is aimed at symptomatic treatment of KS-associated lymphedema in Kenya (Table 1). This study assesses the potential role of locally sourced compression devices compared with traditional elastic stockings, which are significantly more expensive and less accessible.

Four AMC clinical trials have been developed for KS, 2 in collaboration with the AIDS Clinical Trials Group (ACTG; Table 2). The first (ClinicalTrials.gov identifier: NCT01352117) was a prospective, randomized, phase III trial to evaluate the role of early versus delayed oral etoposide in people with mild-to-moderate KS who were initiating ART.^18^ The study enrolled 192 participants—93% from SSA—and although the primary outcome did not differ between arms, time to KS progression (*P* = .021), KS immune response inflammatory syndrome (*P* = .003), and KS response (*P* = .003) favored the early chemotherapy arm. Additional analyses of associations between early chemotherapy and KS immune response inflammatory syndrome and early KS progression are ongoing,^19^ as are studies of baseline prevalence of KSHV inflammatory cytokine syndrome among enrolled participants.^20^

The second collaborative AMC/ACTG study (ClinicalTrials.gov identifier: NCT01435018) was a randomized controlled trial to investigate optimal chemotherapy regimens combined with effective ART for people with advanced AIDS-associated KS.^21^ This study randomly assigned 334 PLWH (96% from SSA) with advanced-stage AIDS-associated KS to receive ART concurrently with open-label chemotherapy—either bleomycin combined with vincristine or oral etoposide (the investigational arms), or paclitaxel (the active control). The study showed that when combined with an effective ART regimen, paclitaxel was superior to both bleomycin combined with vincristine and oral etoposide with respect to progression-free survival, overall response rate, response duration, and a composite of time to progression or death. In addition, paclitaxel was associated with increases in CD4 T-lymphocyte counts, suppression of HIV viremia, and an acceptable adverse event profile. Analyses of pretreatment KS biopsy specimens from this study indicated high expression of WT-1 (Wilms tumor protein) in KS lesions, identifying a potential future therapeutic target in KS,^22^ as well as an association between large numbers of CD8^+^ cells in KS lesions and KSHV gene expression.^23^

Other novel AMC studies include (1) a pilot study (ClinicalTrials.gov identifier: NCT03077451) being conducted in the United States and SSA to evaluate the efficacy of nelfinavir, an HIV-1 protease inhibitor with multiple potential mechanisms of antitumor activity,^24^ in participants with KS, and (2) a multicenter, phase II study (ClinicalTrials.gov identifier: NCT03601806) in SSA of pomalidomide, a member of a class of orally bioavailable medications called immunomodulatory imide drugs with potent anti-inflammatory, antiangiogenic, and immunomodulatory properties that have shown anti-KS activity.^25,26^

### Cervical Dysplasia and Carcinoma

Increased risks of persistent human papillomavirus (HPV) infection and cervical dysplasia/carcinoma among women living with HIV/AIDS (WLWH) were established early during the HIV epidemic.^27^ Although rates of cervical cancer have significantly declined in high-income countries with effective screening programs, the burden in LMICs remains high.^28^ This is largely attributable to limited access to screening and HPV vaccination in LMICs.^29^ WLWH are substantially more likely to have high-risk HPV infection and persistence compared with immunocompetent women, even when receiving ART.^30,31^ Despite ongoing efforts to scale up HPV vaccination, less than 10% of women age 10 to 20 years in LMICs are currently vaccinated.^32^ Moreover, there is evidence that among WLWH, the quadrivalent HPV vaccine, one of the most widely adopted HPV vaccines, is less immunogenic and results in decreased rates of seroconversion compared with HIV-negative women.^33^ Furthermore, there is growing evidence that secondary prevention—particularly cryotherapy—is not as effective in WLWH, who experience high rates of recurrence after treatment.^34,35^ Lastly, as chemoradiation is the standard of care for locally advanced cervical cancer, the high cost and technical expertise needed to deliver this treatment modality present significant challenges in SSA.^36^

All non-AMC trials for cervical disease are focused on detection of dysplasia and prevention of carcinoma (Table 1). NCT03324009 aims to establish the efficacy of a 2-stage screening algorithm—high-risk HPV (hrHPV) and Papanicolau test cotesting followed by visual inspection with acetic acid (VIA) and colposcopy—with evaluation at 1 year to further inform detection guidelines. NCT03931083 is a screening study of Zambian WLWH that tests the accuracy and test characteristics of a novel portable colposcope (Gynocular; Gynius, Stockholm, Sweden) alone and in combination with other screening methods, including VIA and hrHPV identification. NCT04092257 is a study in Malawi in which a same-day screen-and-treat method is piloted in WLWH using self-collected vaginal brush samples and follow-up VIA with thermocoagulation treatment, if indicated. NCT04191967 is a prevention study in Kenyan WLWH that assesses the efficacy of thermocoagulation (Liger) compared with historical controls treated with cryotherapy. Together, these studies target particular needs in SSA, including improved screening algorithms, with more portable and potentially effective devices, to inform optimal prevention and early detection strategies in the region.

AMC has developed 3 trials for cervical dysplasia or cervical cancer in SSA. A recently activated multicenter trial (ClinicalTrials.gov identifier: NCT03284866) will determine if a 9-valent HPV vaccine reduces the high rate of recurrence of cervical high-grade squamous intraepithelial lesions among WLWH in SSA participating in an HPV test-and-treat program. The study will accrue 536 WLWH age 25 years or older who are high-risk HPV positive, as assessed using the GeneXpert hrHPV assay. The trial makes use of a mobile colposcope that integrates digital image capture and Health Insurance Portability and Accountability Act–compliant cloud-based image storage via a smartphone to facilitate central review of colposcopy procedures to ensure quality and consistency across sites and investigators.

AMC also conducted the first prospective clinical trial in WLWH in SSA with locally advanced cervical cancer. The trial (ClinicalTrials.gov identifier: NCT01590017)^37^ demonstrated the feasibility, safety, and efficacy of combined-modality therapy with cisplatin and radiation plus ART in this setting. Of 38 eligible women who initiated study treatment, 31 (82%) completed treatment. One-year progression-free survival was 76.3% (95% CI, 59.4% to 86.9%) and did not significantly differ according to stage at entry (*P* = .581). Participant-reported adherence to ART was high: By 12 months, 93% of participants had an undetectable viral load.

Building on this trial, a subsequent study (ClinicalTrials.gov identifier: NCT03834571), expected to begin accrual in mid-2020, will evaluate the efficacy of adjuvant chemotherapy with paclitaxel and carboplatin for WLWH with locally advanced cervical cancer (International Federation of Gynecology and Obstetrics stage IIB, III, and IVA) after treatment with weekly cisplatin concomitant with radiation. After completion of chemoradiotherapy, eligible participants will be randomly assigned to either adjuvant chemotherapy with paclitaxel and carboplatin or active monitoring. The primary end point is 2-year progression-free survival.

### Lymphoma

Lymphomas are a heterogenous group of diseases. AIDS-defining lymphomas are aggressive B-cell NHLs, including diffuse large-B-cell lymphoma, Burkitt lymphoma, and primary CNS lymphomas, among others. In addition, the risk of other lymphomas, including classic Hodgkin lymphoma, is also increased in the presence of HIV.^12,14^ Like other malignancies discussed above, these lymphomas are often associated with viral infections, most commonly Epstein-Barr virus, which is detected in 30% to 100% of cases, depending on the specific lymphoma subtype, and less commonly KSHV infection.^12^ In SSA, lymphomas are a common malignancy, particular among individuals < 60 years of age.^38^ Moreover, there is evidence showing that even with the increased availability and accessibility of ART, rates of lymphomas are increasing.^39-41^ In high-resource settings, multiagent chemotherapy regimens combined with rituximab and ART have significantly improved overall survival across the wide spectrum of lymphomas, leading to similar outcomes, irrespective of HIV infection status.^42-48^ However, given the difficulties in SSA with cost, logistical complexity, treatment-associated complications, and high levels of supportive care necessary to deliver the aforementioned therapies, implementation of these strategies remains challenging.^9^

The only active HIV lymphoma trial we identified in SSA through our search was a randomized, phase II, multicenter trial (ClinicalTrials.gov identifier: NCT01775475) developed by the AMC to compare an oral chemotherapy regimen with a standard intravenous cyclophosphamide, doxorubicin, vincristine, and prednisone regimen for the treatment of stage III and IV diffuse large B-cell lymphoma. This study built on a previous trial conducted in Uganda and Kenya that demonstrated the oral chemotherapy regimen to be tolerable with an excellent objective response rate of 78%.^49^ As described elsewhere,^50^ AMC-068 closed early because of poor accrual. An analysis of the underlying reasons for premature study closure that will help to guide future AMC lymphoma studies in SSA is the subject of a separate report in this issue.^50^

### Other HIVAMs

Along with the neoplasms discussed above, risks of developing several other virally mediated cancers, including anal, head and neck, vulvar, penile, and hepatocellular cancers, as well as other non–virally derived malignancies, including lung cancers and leukemias, are increased in PLWH.

To our knowledge, only one trial in SSA proposes to study a non–AIDS-defining neoplasm, ocular surface squamous neoplasia (OSSN). OSSN is a relatively rare cancer in Western countries, but shows a markedly increased incidence in SSA. Diagnosis encompasses a spectrum of diseases of the conjunctiva or corneal epithelium, ranging from dysplasia to invasive tumors with intraocular or orbital extension.^51-54^ Risk factors for OSSN include HIV and UV exposure. A role for HPV has also been proposed but not proven.^55^ Given this combination, it is not surprising that there has been a rapid increase in the incidence of OSSN in SSA.^56-58^ As OSSN is one of the first presenting signs of HIV/AIDS in upwards of 50% of cases,^55^ there have been increased efforts to screen for and treat the condition.

The planned AMC study will assess the feasibility of conducting multicenter clinical trials of surgical excision of OSSN in PLWH at AMC sites in Malawi, Uganda, Kenya and Zimbabwe, which have some of the highest rates of OSSN in the world. At each trial site, an ophthalmologist will directly lead the effort, and a pathologist will carry out the initial histologic analysis of the specimen. The study, which is expected to begin in late 2020, includes central pathology review and collection of specimens to evaluate the potential role of HPV in the development of OSSN.

### Other AMC Studies

AMC has supported noninterventional trials in SSA. These include a longitudinal quality-of-life study in patients receiving standard chemotherapy for treatment of HIV-associated KS (ClinicalTrials.gov identifier: NCT03596918) in Tanzania, a study of KSHV gene expression profiles in KS tumor biopsies in Malawi,^59^ and a survey study of HIVAM research and treatment infrastructure at 9 programs in 7 SSA countries that helped inform the choice of AMC’s African trials sites.

## DEVELOPMENT OF AMC INFRASTRUCTURE: EVALUATION, ONBOARDING, CAPACITY, PATHOLOGY, AND TRAINING

In 2010, when the decision was made to expand AMC to SSA, a process was developed to evaluate international sites and integrate them into the broader AMC clinical trials research agenda. The process started with a detailed, initial written application, followed by an onsite audit, and culminated in the activation of introductory noninterventional trials. The initial application evaluated local epidemiology, patient volumes, staff qualifications, research governance, and available equipment to support cancer trials.

### Site Evaluation and Onboarding

Early noninterventional and low-demand trials helped familiarize sites with AMC regulatory requirements, tools, and data collection and quality practices, and helped identify site strengths, opportunities for additional AMC integration, and practical barriers to trial implementation. Regulatory education included Good Clinical Practice and Human Subjects Protection. Robust data-capture templates and quality-control methods were developed by protocol leadership. The AMC Operations and Data Management Center worked with site personnel to develop site implementation plans that included site-specific resources and operational elements—for example, recruitment strategies—to identify and resolve potential implementation barriers.

Beyond this mutual familiarization period, AMC has used targeted development of clinical and laboratory skills, as well as equipment purchases to grow cancer trial infrastructure. Consultants with experience in cancer research pharmacy and nursing procedures were engaged to develop lectures and practical workshops. Site pharmacists reviewed best practices and developed site-specific standard operating procedures for chemotherapy preparation and safe handling, and received on-site evaluation, additional instruction, and trial-specific training in good documentation practices. Site nurses, many of whom had only received informal training in oncology, attended workshops in chemotherapy administration, patient monitoring, and personal protective equipment best practices. Finally, purchases to support AMC clinical trials have ranged from consumables needed for care delivery, such as intravenous fluids, to new technology, including data-sharing devices, such as MobileODT—a handheld, mobile colposcope that allows real-time sharing of results via the Internet for consultations—and laboratory equipment, like GeneXpert, to inform treatment decisions and streamline care by providing real-time results.

### Strengthening Pathology Capacity

Pathologists are a critical component of accurate cancer diagnosis and classification, and treatment success is highly dependent on rigorous knowledge of cancer type, grade, and invasion into relevant structures. Whereas detailed data on the number of pathology laboratories and pathologists in SSA are not available, it is clear there is inadequate access and highly variable standards in SSA.^60,61^ There is a critical lack of pathologists and in-region training opportunities. In SSA, there are typically more than 1 million people per pathologist: 1 pathologist per 1,467,708 people in Uganda, 1 per 1,784,000 in Malawi, 1 per 2,236,000 in Rwanda, 1 per 2,542,333 in Zambia, and 1 per 2,685,200 in Zimbabwe.^62^ Only South Africa and Botswana have relatively adequate coverage: 1 pathologist per 224,897 and 359,333, respectively.^62^ This is in contrast to the United States, where the ratio is around 1 pathologist per 25,000 people.^63^ Pathology laboratories in most of these settings are underfunded and understaffed, and even basic reagents are lacking. Only a handful of laboratories can perform techniques, like immunohistochemistry, that are standard in high-income countries.

Given these limitations, there were significant challenges to conducting cancer clinical trials in SSA. The 2 large collaborative clinical trials performed by ACTG and AMC set a precedent for working with several pathology laboratories in Kenya, Zimbabwe, Malawi, and South Africa. Surveys of site capabilities were conducted to determine where pathology services were available for the site to participate. To build capacity and ensure proper pathologic diagnosis, an extensive external quality assurance program was implemented. The process for credentialing consisted of both a review of the process by which laboratories produced slides for immunohistochemistry and a slide assessment by collaborating pathologists.

Through the AMC NHL study, sites were helped to develop capacity for immunohistochemical staining using CD20 and Ki-67, antibodies that allow for diagnosis of B-cell lymphomas and proliferation rate, respectively. Training, reagents, and technical support were provided through AMC. Digital and in-person case conferences with pathologists from Africa, Brazil, and the United States provided an opportunity to review cases and gain consensus on the inclusion and exclusion of patients on the basis of pathologic diagnosis. Additional growth and sustained best practices are likely only possible with continued internal stakeholder support, coupled with international partnerships, such as with AMC. Of importance, clinical research through AMC in SSA is supported by an SSA regional biospecimen repository established at Stellenbosch University/Tygerberg Hospital in Cape Town, where biospecimens from AMC clinical trials in SSA are collected, processed, and stored or subsequently shipped to AMC-assigned laboratories for additional analyses.^64^

### AMC Fellowships

The AMC fellowship program was initiated in 2014 to augment the human capacity for HIVAM clinical research. This program provides up to 2 years of funding—up to $25,000 per year—to support a mentored clinical, translational, or basic HIVAM-based research project with funds attributable to a fellow’s salary and/or to educational or research efforts. The purpose of the program is to support the next generation of AMC investigators by identifying, recruiting, and fostering early-career investigators to pursue careers in HIVAM. Applications are reviewed annually, and the projects must be aligned with AMC goals and endorsed by the local AMC site principal investigator and a designated research mentor. Fellows are required to attend the semiannual AMC Group Investigators’ meetings, where new and former fellows present either their proposed research plan/progress or study conclusions. Thus far, 31 individuals have received AMC fellowship awards, 29 mentors have been involved in supporting the work of AMC fellows, and 20 awardees have presented their work at the biannual fellow symposium. Of note, 16 of 31 fellowships have been awarded to US or SSA investigators working primarily on projects at SSA sites, reflecting a high degree of interest among early-career investigators to address HIVAM in SSA, many who have gone on to leadership positions within AMC, chair AMC protocols, and successfully compete for peer-reviewed funding.

### Additional Infrastructure Challenges

Effective programmatic application of ART to SSA populations arguably represents one of the most remarkable global public health achievements in recent decades. Although AMC has provided some infrastructure for cancer clinical trials among PLWH in SSA, there remain key operational gaps along the entire spectrum of cancer care that have been largely unaddressed by prior HIV-related investments.^65^ In addition to the gaps in diagnostic pathology mentioned above,^66,67^ trained surgical oncologists are few, although innovative recent solutions to address this exist in SSA.^68,69^ Radiation oncology, with high up-front equipment and maintenance costs, presents unique challenges, with both machines and personnel not present in more than 30 countries.^36^ Even when present, available numbers of treatment units, radiation oncologists, physicists, dosimetrists, and radiation therapists are highly inadequate across SSA to meet predicted demand.^70^ Furthermore, outdated equipment and frequent downtime compound the problems with radiotherapy access.^71^ In addition, medical oncologists are absent in 7 SSA countries, and even where present, average annual workloads are untenable at approximately 3,700 new cancer cases per working oncologist.^72^ These difficulties in delivering cancer care are compounded by deficits in drug availability, oncology nursing, and pharmacist expertise. However, there are good examples of efforts to initiate and improve comprehensive cancer programs in SSA,^73-75^ which have effectively increased access to cancer care among PLWH.^76^ These efforts are essential and ideally should be closely aligned with research initiatives that rigorously define best approaches to prevent and treat HIVAM in SSA. In addition to structural challenges, sociologic challenges remain a barrier to HIV cancer care. To this end, AMC has worked to establish community advisory boards to help bolster and facilitate its trials work. Community advisory boards work to build community capacity through:

1. Education of research teams on local cultural and community norms that may affect research recruitment, entry, and consent
2. Advising the community of the ethical and legal rights of participants
3. Guidance to the research team regarding community-specific risks and burdens from research development to management; and
4. Facilitating accurate information to and from the research group to the study community.^77,78^

## FUTURE DIRECTIONS

Successful global investments in HIV treatment have led to an increasing prevalence of noncommunicable diseases, including HIVAM, among PLWH in SSA. This is similar to high-income countries, where ART availability has led to marked increases in life expectancy and declines in infectious deaths as a result of attributable causes among PLWH. AMC has made important advances in the last decade to begin to fill critical gaps in scientific evidence to guide the management of HIVAM in SSA and build capacity along the cancer continuum, from pathologic diagnosis to cancer therapy to survivorship, to address the rising incidence of cancer among PLWH in SSA. AMC’s comprehensive approach, which involves site and community engagement at all stages of development and execution, should support the long-term sustainability of these advances. As AMC strategizes about the next decade of clinical trials within SSA, the focus will remain on diseases with the highest prevalence in SSA in PLWH, including KS, invasive cervical cancer, and lymphoma. In addition, a variety of other studies will be added, including surgery-focused studies, particularly for OSSN and invasive cervical carcinoma; other disease sites, such as hepatocellular carcinoma; different population groups (ie, pediatric KS); symptom management studies; and behavioral research investigating quality of life and stigma. Through coordinated capacity building and sustained partnerships with SSA institutions and investigators, AMC will continue to advance knowledge in HIVAM by expanding clinical trials through the scope of its portfolio in SSA.
